# Genetic alterations in *LEP* and *ADIPOQ* genes and risk for breast cancer: a meta-analysis

**DOI:** 10.3389/fonc.2023.1125189

**Published:** 2023-05-19

**Authors:** Wei-zhao Peng, Xin Liu, Chao-feng Li, Jin Zhao

**Affiliations:** Department of General Surgery, China-Japan Friendship Hospital, Beijing, China

**Keywords:** breast cancer, genetic alternation, meta-analysis, risk, association

## Abstract

**Introduction:**

Breast cancer has a strong genetic predisposition, and its genetic architecture is not fully understood thus far. In this study, we aimed to perform a meta-analysis to evaluate the association of genetic alterations in *LEP* and *ADIPOQ* genes, as well as their receptor-encoded genes with risk for breast cancer.

**Methods:**

Only published studies conducted in humans and written in English were identified by searching PubMed, SCOPUS, CINAHIL and Embase from their inception to October 2022. Eligibility assessment and data collection were completed independently by two researchers. Statistical analyses were done using the STATA software.

**Results:**

After literature search, 33 publications were eligible for inclusion. Overall, *LEP* gene rs7799039-G allele (odds ratio [OR]: 0.78, 95% confidence interval [CI]: 0.62 to 0.98) and *ADIPOQ* gene rs1501299-T allele (OR: 1.41, 95% CI: 1.06 to 1.88) were associated with the significant risk of breast cancer. In subgroup analyses, differences in menopausal status, obesity, race, study design, diagnosis of breast cancer, genotyping method and sample size might account for the divergent observations of individual studies. Circulating leptin levels were comparable across genotypes of *LEP* gene rs7799039, as well as that of *LEPR* gene rs1137101 (P>0.05). Begg’s funnel plots seemed symmetrical, with the exception of *LEPR* gene rs1137100 and *ADIPOQ* gene rs1501299.

**Discussion:**

Taken together, we found, in this meta-analysis, that *LEP* gene rs7799039 and *ADIPOQ* gene rs1501299 were two promising candidate loci in predisposition to breast cancer risk.

## Introduction

Breast cancer is a leading cause of death in women (1). Global statistics show that the incidence rate of breast cancer was annually increased by 0.5% during the period from 2010 to 2019 (2). Breast cancer is a multifactorial malignancy that has a genetic predisposition (3). Prior studies have demonstrated that the development of mammary carcinoma in the opposite breast of familial patients with unilateral disease was three times higher than that in sporadic patients (4). Recently, a growing number of genome-wide association studies have been conducted to decipher the genetic architecture of breast cancer worldwide (5–9). In spite of great endeavors, deciphering genetic codes of breast cancer is still in its infancy. Evaluating genes with definitive biological function and direct implications in breast carcinogenesis represents a good alternative. Echoing this claim, obesity-related cytokines such as leptin and adiponectin are increasingly recognized as promising candidates in the development of breast cancer (10).

It is widely recognized that obesity is linked to an enhanced risk of tumorigenesis (11). Leptin as an inducer of epithelial-mesenchymal transition was found to promote tumor progression and metastasis (11). Experimental data supported that leptin can influence mammary tumor growth and progression through regulation of autocrine/paracrine factors and by modulating the extracellular matrix composition (12). Clinical evidence showed that women with breast cancer had increased levels of circulating leptin and its receptor (13). Another important obesity-related cytokine, adiponectin, was found to be capable to induce autophagic cell death in breast cancer cells through STK11/LKB1-mediated activation of the AMPK-ULK1 axis (14). There is evidence that circulating adiponectin levels were lower in women with breast cancer than in healthy controls, especially in postmenopausal women (15). Grossmann and Cleary have written an excellent review and highlighted the balance between leptin and adiponectin in the control of mammary tumorigenesis (16). Specifically, imbalance in leptin-adiponectin levels and leptin receptor expression was found to precipitate the progression of triple negative breast cancer (17). Above data collectively support the contributory roles of leptin and adiponectin in the pathogenesis of breast cancer. We thereby hypothesize that genes coding leptin (*LEP*) and adiponectin (*ADIPOQ*) and their receptors are promising candidates in predisposition to breast cancer risk.

To test this hypothesis, we conducted a meta-analysis on genetic alterations in *LEP* and *ADIPOQ* genes as well as their receptor-encoded genes by pooling published summary data, aiming to evaluate their association with risk for breast cancer, as well as circulating leptin and adiponectin levels.

## Methods

### Meta-analysis guideline

The conduct of this meta-analysis complied with the PRISMA (Preferred Reporting Items for Systematic Reviews and Meta-Analyses) guideline (18).

### Search strategy

Only peer-reviewed published studies were retrieved in this meta-analysis by searching PubMed, SCOPUS, CINAHIL and Embase electronic datasets from their inception to October 2022. The key words used for indexing studies in above datasets were formulated from the MeSH (Medical Subject Headings) database, and they are expressed in logistic relations, that is, (“breast cancer” or “breast neoplasm” or “breast tumor” or “breast carcinoma” or “cancer or breast” or “mammary cancer”) and (“leptin” or “lep” or “leptin receptor” or “adiponectin” or “ADIPOQ” or “adiponectin receptor” or “ADIPOQR”) and (“polymorphism” or “variant” or “mutation” or “mutant” or “SNP” or “allele” or “genotype”). The search process was completed by two researchers (X.L. and C.L.) independently. Search results from different datasets were managed by the ENDNOTE software version X9.3.3, and duplicate records were deleted.

In addition, potential missing studies were complemented by checking the references of reviews, meta-analyses and major original articles in search results.

### Eligibility criteria

Eligible studies were expected to meet all five inclusion criteria (1): breast cancer as clinical outcome (2); involvement of both breast cancer patients and control participants (3); complete genetic data (genotypes or alleles or effect sizes) of any genetic alteration in *LEP* or *ADIPOQ* genes or their receptor-encoded genes between patients with breast cancer and controls or mean or median values of circulating leptin or adiponectin levels for single genotypes or their combination (4); publication using English language (5); valid diagnosis of breast cancer.

Meanwhile, other forms of publications such as comment, editorial, perspective, letter to the editor and case report/series were not covered.

### Data collection

Collection of necessary data from eligible studies was independently conducted by two researchers (X.L. and C.L.). Items of data covered surname of first author, year of publication, study design, race, country where study participants resided in, menopause status, source of control participants, factors matched between patients and controls, genotyping method, diagnostic criteria of breast cancer, sample size, chronological age, age at menarche, age of first delivery, nulliparous, percentage of ER, PR and Her-2 of patients with breast cancer, height, weight, body mass index, cigarette smoking, alcohol drinking, family history of breast cancer and genotypes of genetic alteration between patients and controls.

If there was disagreement between the two researchers, original article was assessed, and if necessary, a third researcher (W.P.) was involved.

### Data analyses

Summary data from identified eligible studies were pooled by the Stata software version 15.0. To derive a sufficient power to detect significance, a minimum number of eligible studies was set at 3 for genetic alterations analyzed in this study. The association between genetic alterations and breast cancer was expressed as odds ratio (OR) and 95% confidence interval (95% CI). The association between genetic alterations and circulating leptin or adiponectin levels was expressed as standardized mean difference (SMD) and its 95% CI. Effect-size estimates were generated using the DerSimonian-Laird method and under the random-effects model. Heterogeneity between studies was justified by using a percent, inconsistency index (*I* ^2^), and *I* ^2^ over 50% or the probability of associated χ^2^ test less than 0.1 was indicative of statistical significance. Exploring sources of heterogeneity was implemented by using subgroup analyses according to categorical items of interest. Contribution of each study to overall OR was illustrated by sensitivity analyses.

Publication bias was judged by the Begg’s funnel plot and Egger’s linear regression tests from visual and statistical aspects, respectively. In the case of evident publication bias, the trim-and-fill method was used to take theoretically missing studies into consideration when estimating effect-size estimates.

## Results

### Eligible studies

**Figure 1:**
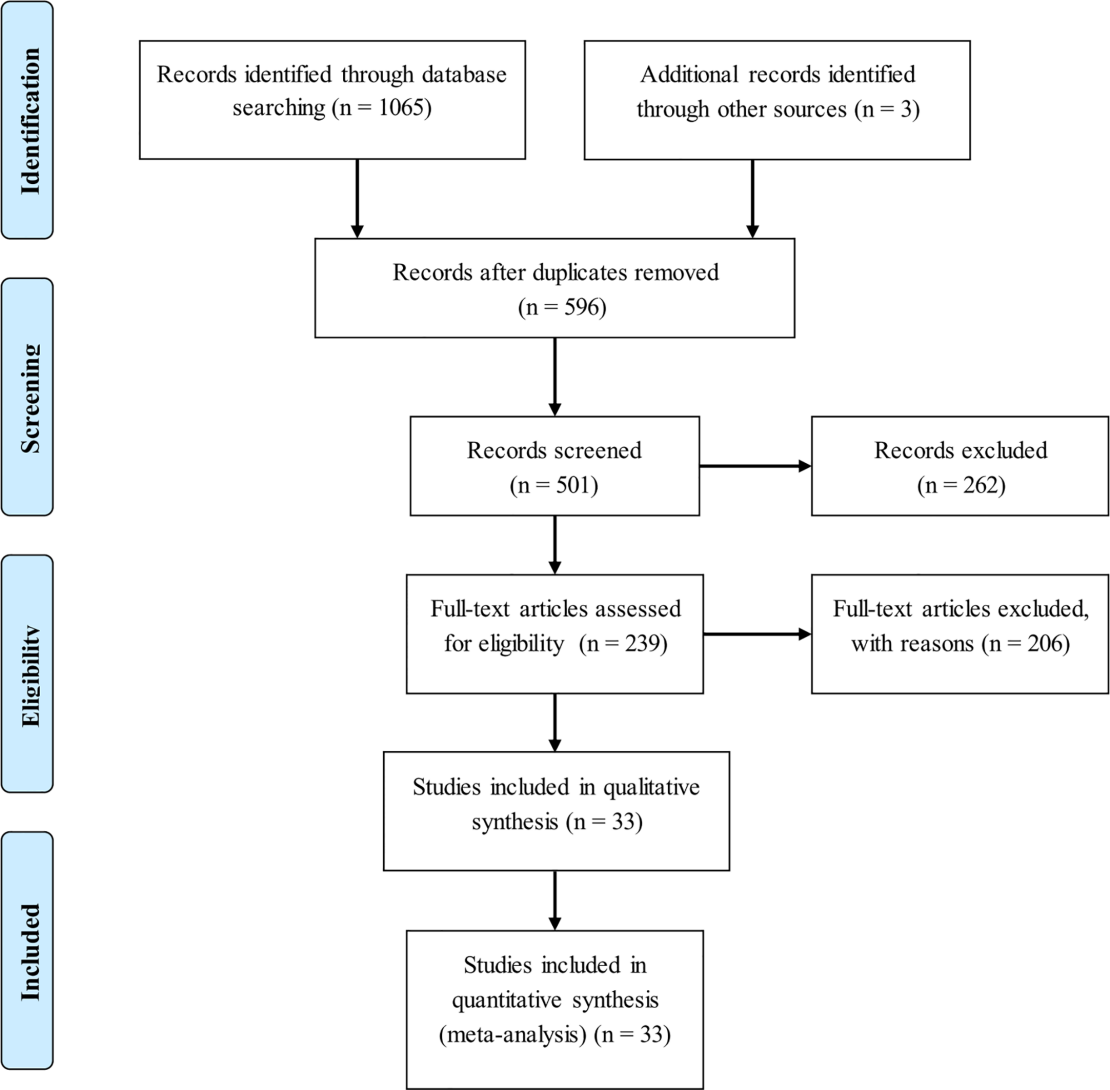
Flow chart illustrating the selection of qualified studies.

Initial search of 4 public datasets identified a total of 596 publications after deleting duplicates. Only 33 of these publications were eligible for inclusion (10, 19–50). The selection process of eligible articles was displayed in the form of flow diagram (**Figure 1**). In the case of publications containing more than one group, each group was treated separately. Finally, 55 studies were meta-analyzed for the association between 5 genetic alterations in 3 genes (*LEP*, leptin receptor [*LEPR*] and *ADIPOQ*) and breast cancer risk, 4 studies for the association between rs7799039 genotypes and circulating leptin levels, and 8 studies for the association between rs1137101 genotypes and circulating leptin levels.

The baseline characteristics of 55 studies in this meta-analysis are presented in **Table 1**.

**Table 1:** Characteristics of 33 publications in this meta-analysis.

Author	Year	Menopause	Country	Race	Study design	Source of controls	Matched items	Genotyping method	Diagnosis of breast cancer
Li et al	2022	pre and post	China	East Asian	Retrospective	Population	age	Array	histologically-confirmed
Li et al. (ER+)	2022	pre and post	China	East Asian	Retrospective	Population	age	Array	histologically-confirmed
Li et al. (ER-)	2022	pre and post	China	East Asian	Retrospective	Population	age	Array	histologically-confirmed
Li et al. (Normal)	2022	pre and post	China	East Asian	Retrospective	Population	age	Array	histologically-confirmed
Li et al. (Obese)	2022	pre and post	China	East Asian	Retrospective	Population	age	Array	histologically-confirmed
Atoum et al	2022	pre and post	Jordan	Middle Eastern	Retrospective	Hospital	NA	RFLP	pathology-based
Atoum et al. (Normal)	2022	pre and post	Jordan	Middle Eastern	Retrospective	Hospital	NA	RFLP	pathology-based
Atoum et al. (Obese)	2022	pre and post	Jordan	Middle Eastern	Retrospective	Hospital	NA	RFLP	pathology-based
Özgöz et al. (ER+)	2021	post	Turkey	Middle Eastern	Retrospective	Hospital	NA	Array	hospital-diagnosed
Hołysz	2021	post	Polish	European	Retrospective	Population	NA	RFLP	hospital-diagnosed
Hołysz (ER+)	2021	post	Polish	European	Retrospective	Population	NA	RFLP	hospital-diagnosed
Hołysz (ER-)	2021	post	Polish	European	Retrospective	Population	NA	RFLP	hospital-diagnosed
Mahmoud et al. (Obese)	2020	post	Ezype	Middle Eastern	Retrospective	Population	BMI	RFLP	hospital-diagnosed
Cerda-Flores et al	2020	pre and post	Mexico	Hispanic	Retrospective	Hospital	NA	TaqMan	histologically-confirmed
Pasha et al. (Obese)	2019	pre and post	Ezype	Middle Eastern	Retrospective	Hospital	age	RFLP	histologically-confirmed
Macias-Gomez et al	2019	pre and post	Jalisco	Hispanic	Retrospective	Hospital	NA	RFLP	histologically-confirmed
Geriki et al	2019	pre and post	India	East Asian	Prospective	Hospital	age	RFLP	hospital-diagnosed
Liu et al. (premeno)	2018	pre	China	East Asian	Prospective	Hospital	residence	Array	histologically-confirmed
Liu et al. (postmeno)	2018	post	China	East Asian	Prospective	Hospital	residence	Array	histologically-confirmed
Rodrigo et al	2017	pre and post	Sri Lanka	East Asian	Retrospective	Hospital	age, BMI, menopausal status	SNaPshot	hospital-diagnosed
Rodrigo et al. (premeno)	2017	pre and post	Sri Lanka	East Asian	Retrospective	Hospital	age, BMI, menopausal status	SNaPshot	hospital-diagnosed
Rodrigo et al. (postmeno)	2017	pre and post	Sri Lanka	East Asian	Retrospective	Hospital	age, BMI, menopausal status	SNaPshot	hospital-diagnosed
El-Hussiny et al	2017	pre and post	Ezype	Middle Eastern	Retrospective	Hospital	NA	RFLP	hospital-diagnosed
Khandouzi et al	2016	pre and post	India	East Asian	Retrospective	Hospital	NA	RFLP	hospital-diagnosed
Erbay et al	2016	pre and post	Turkey	Middle Eastern	Retrospective	Hospital	NA	RFLP	histologically-confirmed
Rostami et al	2015	pre and post	Iran	Middle Eastern	Retrospective	Hospital	age, sex	RFLP	hospital-diagnosed
Mohammadzadeh et al	2015	pre and post	Iran	Middle Eastern	Retrospective	Hospital	age, BMI, menopausal status	RFLP	hospital-diagnosed
Mohammadzadeh et al. (premeno)	2015	pre and post	Iran	Middle Eastern	Retrospective	Hospital	age, BMI, menopausal status	RFLP	hospital-diagnosed
Mohammadzadeh et al. (postmeno)	2015	pre and post	Iran	Middle Eastern	Retrospective	Hospital	age, BMI, menopausal status	RFLP	hospital-diagnosed
Mahmoudi et al	2015	pre and post	Iran	Middle Eastern	Retrospective	Hospital	NA	RFLP	pathology-based
Karakus et al	2015	pre and post	Turkey	Middle Eastern	Retrospective	Hospital	NA	RFLP	histologically-confirmed
Mohammadzadeh et al	2014	pre and post	Iran	Middle Eastern	Retrospective	Hospital	age	RFLP	hospital-diagnosed
Robles et al. (obese)	2013	pre and post	Mexico	Hispanic	Retrospective	Hospital	NA	RFLP	hospital-diagnosed
Robles et al. (obese, premeno)	2013	pre and post	Mexico	Hispanic	Retrospective	Hospital	NA	RFLP	hospital-diagnosed
Robles et al. (obese, postmeno)	2013	pre and post	Mexico	Hispanic	Retrospective	Hospital	NA	RFLP	hospital-diagnosed
Kaklamani et al. (AA)	2013	post	USA	American	Prospective	Population	NA	Array	hospital-diagnosed
Kaklamani et al. (Hispanics)	2013	post	USA	American	Prospective	Population	NA	Array	hospital-diagnosed
Kim et al	2012	pre and post	Korea	East Asian	Retrospective	Hospital	age	Array	hospital-diagnosed
Gu et al	2012	pre	USA (Caucasian)	American	Prospective	Population	age	Array	hospital-diagnosed
Nyante et al	2011	pre and post	USA	American	Prospective	Population	age, race	Array	histologically-confirmed
Cleveland et al	2010	pre and post	USA	American	Prospective	Population	age	RFLP	histologically-confirmed
Cleveland et al. (permeno, BMI<30)	2010	pre	USA	American	Prospective	Population	age	RFLP	histologically-confirmed
Cleveland et al. (permeno, BMI>=30)	2010	pre	USA	American	Prospective	Population	age	RFLP	histologically-confirmed
Cleveland et al. (postmeno, BMI<30)	2010	post	USA	American	Prospective	Population	age	RFLP	histologically-confirmed
Cleveland et al. (postmeno, BMI>=30)	2010	post	USA	American	Prospective	Population	age	RFLP	histologically-confirmed
Teras et al	2009	post	USA	American	Prospective	Population	age, race and blood draw date	Array	hospital-diagnosed
Okobia et al	2008	pre and post	Nigeria	African	Prospective	Hospital	age	RFLP	hospital-diagnosed
Okobia et al. (premeno)	2008	pre	Nigeria	African	Prospective	Hospital	age	RFLP	hospital-diagnosed
Okobia et al. (postmeno)	2008	post	Nigeria	African	Prospective	Hospital	age	RFLP	hospital-diagnosed
Kaklamani et al	2008	pre and post	USA	American	Retrospective	Hospital	gender, region	Array	hospital-diagnosed
Han et al	2008	pre and post	China	East Asian	Retrospective	Hospital	age, region, race	RFLP	pathology-based
Liu et al	2007	pre and post	China	East Asian	Retrospective	Population	age	RFLP	hospital-diagnosed
Gallicchio et al	2007	pre and post	USA	American	Prospective	Population	NA	TaqMan	hospital-diagnosed
Woo et al	2006	pre and post	Korea	East Asian	Retrospective	Population	age	Sequencing	hospital-diagnosed
Snoussi et al	2006	pre and post	Tunisia	Middle Eastern	Retrospective	Population	NA	RFLP	histologically-confirmed

NA, Not available.

### Overall association analyses

**Figure 2:**
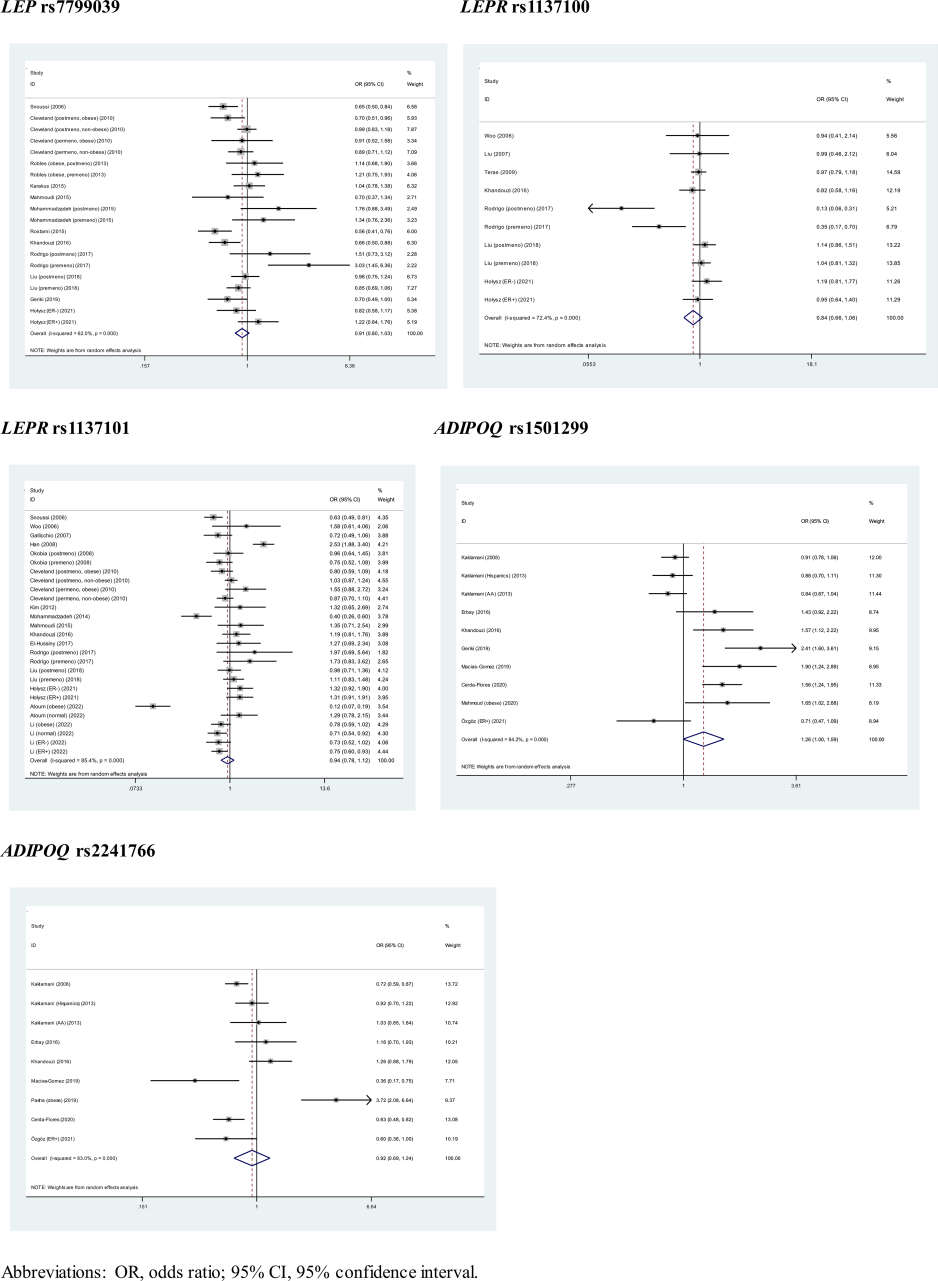
Pooled estimates of 5 genetic alterations associated with breast cancer under allele mode of inheritance.

The association of 5 genetic alterations with breast cancer risk was displayed in the form of forest plots under allele mode of inheritance (**Figure 2**). Overall, *LEP* gene rs7799039-G allele and *LEPR* gene rs1137100-A allele were associated with reduced breast cancer risk relative to the corresponding reference alleles, and the risk was close to statistical significance. By contrast, *ADIPOQ* gene rs1501299-T allele increased breast cancer risk significantly by 26% (OR: 1.26, 95% CI: 1.00 to 1.59) relative to the corresponding G allele. The *I* ^2^ ranged from 62% to 85.4%, denoting the moderate-strong evidence of heterogeneity between studies.

Besides allele mode, pooled estimates under dominant and genotype modes of inheritance are shown in **Supplementary Figure 1** and **Supplementary Figure 2**, respectively. Under dominant mode, the protective effects of *LEP* gene rs7799039 GG plus GA genotypes and *LEPR* gene rs1137100 AA plus AG genotypes on breast cancer risk dwindled, and the risk conferred by *ADIPOQ* gene rs1501299 TT plus TG genotypes was enhanced, with OR of 1.41 (95% CI: 1.06 to 1.88). Under genotype mode, *LEP* gene rs7799039-GG was associated with a 22% reduced risk of breast cancer significantly (OR: 0.78, 95% CI: 0.62 to 0.98), and no significance was detected for the other comparisons.

### Subgroup analyses by menopause and obesity

The association of 5 genetic alterations with breast cancer stratified by menopause and obesity is summarized in **Table 2**.

**Table 2:** Subgroup association analyses of 5 genetic alterations with breast cancer by menopause and obesity.

Genetic alterations	Subgroups	N	Allele mode (R *vs.* W)					Dominant mode (RR plus RW *vs.* WW)					Genotype mode (RR *vs.* WW)				
			OR	95% CI	P	*I* ^2^	P_het_	OR	95% CI	P	*I* ^2^	P_het_	OR	95% CI	P	*I* ^2^	P_het_
Menopausal																	
***LEP* rs7799039**	Both	12	0.96	.76 - 1.22	0.755	73.39%	<0.001	1.02	.69 - 1.51	0.920	75.14%	<0.001	0.77	.51 - 1.15	0.200	51.23%	0.020
	Postmenopausal	4	0.95	.81 - 1.11	0.497	40.24%	0.170	0.87	.68 - 1.10	0.244	32.39%	0.218	0.93	.65 - 1.34	0.694	45.53%	0.138
	Premenopausal	3	0.87	.75 - 1.02	0.076	0%	0.959	0.81	.65 - 1.01	0.055	0%	0.913	0.75	.53 - 1.07	0.114	0%	0.991
***LEPR* rs1137100**	Both	5	0.53	.27 - 1.02	0.058	80.30%	<0.001	0.56	.26 - 1.22	0.142	68.24%	0.013	0.23	.07 -.82	0.024	63.02%	0.044
	Postmenopausal	3	1.01	.88 - 1.17	0.848	0%	0.652	1.25	.94 - 1.67	0.125	0%	0.903	1.10	.64 - 1.89	0.725	0%	0.770
	Premenopausal	1	1.04	.81 - 1.32	0.782	NA	NA	1.02	.77 - 1.35	0.889	NA	NA	1.22	.55 - 2.67	0.626	NA	NA
***LEPR* rs1137101**	Both	13	1.01	.71 - 1.44	0.971	89.79%	<0.001	0.96	.66 - 1.41	0.844	81.01%	<0.001	1.09	.49 - 2.43	0.827	87.79%	<0.001
	Postmenopausal	5	1.01	.89 - 1.13	0.921	0%	0.479	0.99	.78 - 1.26	0.959	38.07%	0.167	1.02	.78 - 1.33	0.884	0%	0.444
	Premenopausal	4	0.97	.77 - 1.23	0.824	50.80%	0.107	0.97	.77 - 1.23	0.813	12.38%	0.331	1.01	.55 - 1.86	0.969	53.27%	0.093
***ADIPOQ* rs1501299**	Both	6	1.53	1.11 - 2.11	0.010	85.71%	<0.001	1.65	1.17 - 2.34	0.005	80.19%	<0.001	1.94	.94 - 4.01	0.071	81.38%	<0.001
	Postmenopausal	4	0.92	.72 - 1.18	0.523	60.57%	0.055	1.09	.70 - 1.69	0.700	76.76%	0.005	0.63	.39 - 1.02	0.061	38.86%	0.195
***ADIPOQ* rs2241766**	Both	6	0.98	.63 - 1.52	0.919	88.67%	<0.001	1.02	.63 - 1.64	0.953	87.43%	<0.001	0.73	.29 - 1.79	0.486	71.19%	0.004
	Postmenopausal	3	0.86	.66 - 1.13	0.278	25.77%	0.260	0.81	.57 - 1.16	0.255	45.58%	0.159	0.92	.44 - 1.92	0.822	0%	0.795
Obesity																	
***LEP* rs7799039**	NA	7	1.02	.79 - 1.33	0.877	77.40%	<0.001	0.99	.67 - 1.46	0.960	78.79%	<0.001	1.01	.59 - 1.74	0.964	64.78%	0.009
	Normal	3	0.89	.75 - 1.06	0.202	35.74%	0.211	0.78	.63 -.98	0.033	0%	0.995	0.78	.57 - 1.06	0.113	23.70%	0.270
	Obese	3	0.93	.63 - 1.36	0.689	67.74%	0.045	0.90	.46 - 1.75	0.755	64.75%	0.059	0.86	.38 - 1.92	0.708	68.13%	0.043
	Overweight	3	0.75	.50 - 1.12	0.156	83.23%	0.003	0.70	.36 - 1.34	0.275	81.80%	0.004	0.63	.31 - 1.28	0.204	76.81%	0.013
***LEPR* rs1137100**	NA	5	0.79	.53 - 1.17	0.242	86.48%	<0.001	0.78	.45 - 1.36	0.374	74.68%	0.003	0.50	.13 - 1.93	0.313	79.53%	0.002
	Normal	1	0.99	.46 - 2.13	0.972	NA	NA	1.28	.51 - 3.22	0.602	NA	NA	0.47	.08 - 2.75	0.401	NA	NA
	Overweight	2	0.92	.73 - 1.14	0.438	0%	0.413	1.02	.61 - 1.71	0.939	49.36%	0.160	0.97	.51 - 1.84	0.920	29.68%	0.233
**LEPR rs1137101**	NA	9	0.98	.78 - 1.21	0.821	61.02%	0.009	0.96	.72 - 1.29	0.806	55.09%	0.023	0.99	.56 - 1.76	0.965	62.79%	0.009
	Normal	4	0.92	.75 - 1.13	0.418	59.01%	0.062	1.03	.68 - 1.55	0.907	80.37%	0.002	0.92	.70 - 1.21	0.552	0%	0.573
	Obese	5	0.68	.33 - 1.40	0.293	93.76%	<0.001	0.57	.25 - 1.29	0.177	91.02%	<0.001	0.76	.21 - 2.68	0.667	87.94%	<0.001
	Overweight	4	1.10	.55 - 2.20	0.787	94.06%	<0.001	1.21	.68 - 2.14	0.513	79.95%	0.002	1.01	.23 - 4.45	0.985	92.64%	<0.001
***ADIPOQ* rs1501299**	NA	5	1.25	.70 - 2.22	0.457	97.26%	<0.001	2.70	.30 - 24.18	0.375	99.66%	<0.001	1.04	.64 - 1.68	0.881	72.39%	0.006
	Normal	1	2.41	1.60 - 3.61	<0.001	NA	NA	2.63	1.58 - 4.40	<0.001	NA	NA	5.14	1.90 - 13.90	0.001	NA	NA
	Obese	1	1.65	1.02 - 2.68	0.043	NA	NA	3.76	1.69 - 8.40	0.001	NA	NA	NA	NA	NA	NA	NA
	Overweight	3	1.29	.74 - 2.26	0.370	83.28%	0.003	1.54	.98 - 2.41	0.061	56.85%	0.099	1.01	.20 - 5.18	0.991	85.60%	0.001
***ADIPOQ* rs2241766**	NA	5	0.81	.67 -.99	0.043	52.82%	0.076	0.81	.66 - 1.00	0.051	45.18%	0.121	0.57	.29 - 1.14	0.113	50.80%	0.087
	Obese	1	3.72	2.08 - 6.64	<0.001	NA	NA	4.05	2.10 - 7.81	<0.001	NA	NA	6.22	1.31 - 29.60	0.022	NA	NA
	Overweight	3	0.68	.33 - 1.41	0.300	82.98%	0.003	0.66	.28 - 1.54	0.330	84.30%	0.002	0.88	.41 - 1.88	0.735	0%	0.438

R, risk allele; W, wild allele; OR, odds ratio; 95% CI, 95% confidence interval; NA, not available.

By menopausal status, *LEPR* gene rs1137100-AA genotype carriers conferred a significantly reduced risk of breast cancer compared with GG genotype carriers (OR: 0.23, 95% CI: 0.07 to 0.82) in both premenopausal and postmenopausal women. For *ADIPOQ* gene rs1501299, the risk for breast cancer was significant under both allele (OR: 1.53, 95% CI: 1.11 to 2.11) and dominant (OR: 1.65, 95% CI: 1.17 to 2.34) modes of inheritance in both premenopausal and postmenopausal women.

By obesity, the association of *LEP* gene rs7799039 GG plus GA genotypes with breast cancer was substantiated in normal-weight women (OR: 0.78, 95% CI: 0.63 to 0.98).

### Subgroup analyses by other features

**Table 3** shows the subgroup association of 5 genetic alterations with breast cancer stratified by other features of interest. For *LEP* rs7799039, the association was significant in studies with prospective design, with histologically-confirmed breast cancer, and involving sample size exceeding 300 under three genetic modes of inheritance. For *ADIPOQ* rs1501299, significance was noticed in women from East Asia, in studies involving hospital-sourced controls, in studies adopting RFLP technique, and in studies with histologically-confirmed breast cancer under both allele and dominant modes.

**Table 3:** Subgroup association analyses of 5 genetic alterations with breast cancer by other features.

Subgroups		N	Allele mode (R *vs.* W)					Dominant mode (RR plus RW *vs.* WW)					Genotype mode (RR *vs.* WW)				
			OR	95% CI	P	*I* ^2^	P_het_	OR	95% CI	P	*I* ^2^	P_het_	OR	95% CI	P	*I* ^2^	P_het_
*LEP* rs7799039																	
Race	East Asian	6	0.95	.72 - 1.25	0.713	73.18%	0.002	1.02	.71 - 1.48	0.909	70.63%	0.004	0.76	.47 - 1.21	0.247	45.95%	0.099
	Middle Eastern	6	0.87	.63 - 1.20	0.395	74.22%	0.002	0.79	.46 - 1.36	0.390	77.12%	0.001	0.69	.38 - 1.27	0.237	57.85%	0.037
	Others	2	1.18	.83 - 1.66	0.362	0%	0.870	1.51	.81 - 2.81	0.194	0%	0.871	1.46	.71 - 3.02	0.309	0%	0.876
	Western	6	0.91	.80 - 1.04	0.184	21.13%	0.275	0.87	.67 - 1.11	0.263	26.57%	0.235	0.83	.65 - 1.06	0.128	7.64%	0.368
Study design	Prospective	7	0.89	.81 -.98	0.013	0.43%	0.420	0.81	.70 -.93	0.003	0%	0.961	0.78	.62 -.97	0.025	6.88%	0.375
	Retrospective	13	0.99	.80 - 1.24	0.961	72.64%	<0.001	1.10	.75 - 1.61	0.617	75.05%	<0.001	0.86	.58 - 1.27	0.438	52.30%	0.014
Control source	Hospital	13	0.97	.80 - 1.18	0.761	68.23%	<0.001	1.02	.77 - 1.35	0.890	69.26%	<0.001	0.85	.61 - 1.18	0.329	39.71%	0.069
	Population	7	0.86	.735 - 1.01	0.060	50.62%	0.059	0.80	.60 - 1.07	0.134	47.57%	0.076	0.72	.52 - 1.01	0.054	48.79%	0.069
Matched	NA	8	0.88	.72 - 1.08	0.218	56.86%	0.023	0.91	.63 - 1.33	0.635	58.98%	0.017	0.82	.54 - 1.26	0.360	55.84%	0.027
	Yes	12	0.93	.79 - 1.11	0.439	67.01%	<0.001	0.92	.72 - 1.19	0.557	67.35%	<0.001	0.76	.58 -.99	0.039	30.25%	0.150
Genotyping method	Array	4	1.19	.81 - 1.75	0.370	74.71%	0.008	1.31	.78 - 2.20	0.315	79.21%	0.002	1.06	.62 - 1.82	0.821	19.42%	0.293
	RFLP	16	0.87	.76 -.99	0.039	58.48%	0.002	0.85	.68 - 1.07	0.161	56.96%	0.003	0.74	.57 -.94	0.015	42.80%	0.036
Diagnosis of BC	Histologically	9	0.87	.78 -.97	0.011	29.49%	0.183	0.79	.68 -.91	0.001	5.65%	0.388	0.77	.59 -.99	0.041	32.94%	0.154
	Non-histologically	11	1.04	.80 - 1.35	0.775	74.10%	<0.001	1.27	.84 - 1.91	0.257	74.35%	<0.001	0.87	.57 - 1.33	0.512	48.94%	0.033
Sample size	Total sample size <300	11	1.11	.89 - 1.40	0.349	53.30%	0.018	1.35	.97 - 1.88	0.079	49.86%	0.030	1.02	.72 - 1.44	0.929	8.77%	0.361
	Total sample size >300	9	0.81	.70 -.93	0.002	62.74%	0.006	0.72	.60 -.87	<0.001	44.68%	0.071	0.67	.51 -.89	0.006	54.16%	0.026
*LEPR* rs1137100																	
Race	East Asian	7	0.70	.47 - 1.04	0.077	80.53%	<0.001	0.86	.60 - 1.25	0.436	63.25%	0.012	0.45	.19 - 1.04	0.061	64.83%	0.014
	Western	3	1.00	.85 - 1.18	0.994	0%	0.618	1.88	.97 - 3.66	0.062	0%	0.558	1.66	.83 - 3.29	0.150	0%	0.539
Study design	Prospective	3	1.03	.90 - 1.17	0.720	0%	0.658	1.10	.89 - 1.36	0.379	0%	0.722	1.04	.57 - 1.90	0.899	0%	0.826
	Retrospective	7	0.68	.44 - 1.05	0.082	78.44%	<0.001	0.83	.44 - 1.58	0.572	68.72%	0.004	0.51	.18 - 1.44	0.204	74.20%	0.002
Control source	Hospital	5	0.63	.39 - 1.02	0.059	86.99%	<0.001	0.77	.48 - 1.24	0.284	75.03%	0.003	0.43	.16 - 1.13	0.086	71.71%	0.007
	Population	5	1.00	.85 - 1.16	0.966	0%	0.912	1.42	.89 - 2.26	0.139	0%	0.596	1.40	.74 - 2.66	0.300	0%	0.402
Matched	NA	3	0.96	.77 - 1.19	0.709	0%	0.370	1.35	.65 - 2.82	0.425	63.77%	0.063	1.23	.56 - 2.69	0.606	49.24%	0.139
	Yes	7	0.74	.52 - 1.05	0.096	80.38%	<0.001	0.85	.54 - 1.33	0.472	61.08%	0.017	0.42	.15 - 1.14	0.089	65.26%	0.013
Genotyping method	Array	6	0.71	.49 - 1.04	0.082	83.64%	<0.001	0.77	.45 - 1.29	0.316	67.13%	0.009	0.39	.12 - 1.31	0.127	72.09%	0.006
	RFLP	4	0.96	.78 - 1.18	0.712	0%	0.574	1.27	.75 - 2.17	0.378	47.27%	0.128	1.08	.54 - 2.18	0.822	38.50%	0.181
Diagnosis of BC	Histologically	2	1.08	.90 - 1.30	0.430	0%	0.625	1.10	.89 - 1.36	0.376	0%	0.422	1.05	.56 - 1.94	0.890	0%	0.539
	Non-histologically	8	0.74	.53 - 1.03	0.073	76.21%	<0.001	0.84	.46 - 1.55	0.586	63.51%	0.008	0.55	.21 - 1.41	0.212	69.10%	0.004
Sample size	Total sample size <300	6	0.64	.36 - 1.13	0.122	82.04%	<0.001	0.76	.31 - 1.87	0.550	73.51%	0.002	0.45	.11 - 1.79	0.257	79.34%	0.001
	Total sample size >300	4	1.00	.88 - 1.13	0.933	0%	0.527	1.03	.86 - 1.25	0.726	0%	0.538	0.89	.54 - 1.47	0.659	0%	0.768
*LEPR* rs1137101																	
Race	East Asian	12	1.10	.85 - 1.42	0.482	82.69%	<0.001	1.03	.81 - 1.32	0.791	73.30%	<0.001	1.33	.68 - 2.62	0.411	71.79%	<0.001
	Middle Eastern	6	0.63	.32 - 1.23	0.172	92.57%	<0.001	0.69	.23 - 2.12	0.518	91.44%	<0.001	0.61	.20 - 1.85	0.380	86.45%	<0.001
	Others	2	0.83	.64 - 1.10	0.191	0%	0.376	0.77	.49 - 1.21	0.259	0%	0.643	0.69	.39 - 1.21	0.191	0%	0.404
	Western	7	1.01	.85 - 1.20	0.909	53.33%	0.045	0.96	.74 - 1.25	0.757	49.69%	0.064	1.00	.72 - 1.40	0.997	48.78%	0.069
Study design	Prospective	9	0.94	.84 - 1.05	0.278	21.40%	0.253	0.92	.78 - 1.08	0.302	21.86%	0.249	0.85	.65 - 1.10	0.214	21.20%	0.254
	Retrospective	18	0.95	.71 - 1.27	0.714	89.78%	<0.001	0.95	.68 - 1.33	0.766	85.33%	<0.001	1.04	.55 - 1.95	0.907	85.04%	<0.001
Control source	Hospital	14	0.986	.67 - 1.46	0.944	90.78%	<0.001	1.01	.64 - 1.58	0.969	86.48%	<0.001	1.21	.53 - 2.75	0.654	86.43%	<0.001
	Population	13	0.88	.76 - 1.01	0.060	62.62%	0.001	0.82	.70 -.96	0.015	47.33%	0.030	0.83	.62 - 1.10	0.190	47.03%	0.036
Matched	NA	9	0.85	.54 - 1.35	0.496	91.06%	<0.001	0.92	.46 - 1.83	0.810	89.54%	<0.001	0.86	.41 - 1.83	0.699	84.39%	<0.001
	Yes	18	0.97	.81 - 1.16	0.736	80.28%	<0.001	0.93	.77 - 1.11	0.402	65.99%	<0.001	1.01	.67 - 1.54	0.953	73.42%	<0.001
Genotyping method	Array	11	0.88	.76 - 1.03	0.117	45.24%	0.051	0.84	.70 – 1.00	0.048	42.97%	0.063	0.88	.57 - 1.35	0.546	22.61%	0.235
	RFLP	16	0.92	.70 - 1.22	0.569	90.44%	<0.001	0.92	.64 - 1.32	0.655	85.79%	<0.001	0.93	.57 - 1.53	0.780	85.77%	<0.001
Diagnosis of BC	Histologically	15	0.87	.68 - 1.11	0.256	90.10%	<0.001	0.86	.65 - 1.14	0.287	86.41%	<0.001	0.91	.55 - 1.52	0.723	83.81%	<0.001
	Non-histologically	12	1.04	.81 - 1.34	0.775	68.36%	<0.001	1.00	.73 - 1.37	0.986	50.91%	0.021	1.00	.58 - 1.73	0.998	67.17%	0.001
Sample size	Total sample size <300	14	0.99	.67 - 1.47	0.964	87.87%	<0.001	1.08	.61 - 1.92	0.792	84.38%	<0.001	1.14	.56 - 2.33	0.719	82.24%	<0.001
	Total sample size >300	13	0.92	.76 - 1.10	0.347	83.05%	<0.001	0.88	.73 - 1.06	0.169	72.82%	<0.001	0.87	.57 - 1.33	0.521	75.81%	<0.001
*ADIPOQ* rs1501299																	
Race	East Asian	2	1.92	1.27 - 2.91	0.002	59.17%	0.118	2.15	1.57 - 2.96	<0.001	0%	0.330	2.45	.51 - 11.86	0.266	72.26%	0.058
	Middle Eastern	3	1.18	.70 - 1.98	0.534	74.93%	0.019	1.66	.81 - 3.39	0.167	74.21%	0.021	0.77	.09 - 6.36	0.810	86.01%	0.008
	Others	2	1.63	1.33 - 1.99	<0.001	0%	0.420	1.79	1.38 - 2.32	<0.001	0%	0.770	2.48	1.55 - 3.96	<0.001	0%	0.337
	Western	3	0.88	.79 -.99	0.029	0%	0.836	0.89	.77 - 1.03	0.122	0%	0.585	0.72	.55 -.94	0.016	0%	0.931
Study design	Prospective	3	1.17	.70 - 1.96	0.540	90.71%	<0.001	1.17	.65 - 2.10	0.602	88.14%	<0.001	1.29	.50 - 3.34	0.599	84.40%	0.002
	Retrospective	7	1.31	.99 - 1.72	0.057	81.09%	<0.001	1.56	1.12 - 2.17	0.009	77.36%	<0.001	1.24	.60 - 2.55	0.567	80.99%	<0.001
Control source	Hospital	7	1.38	1.02 - 1.88	0.039	85.52%	<0.001	1.54	1.12 - 2.12	0.008	77.68%	<0.001	1.50	.73 - 3.089	0.269	82.90%	<0.001
	Population	3	0.99	.74 - 1.33	0.939	68.86%	0.040	1.18	.66 - 2.10	0.575	84.49%	0.002	0.75	.51 - 1.08	0.121	0%	0.767
Matched	NA	7	1.18	.90 - 1.56	0.229	82.49%	<0.001	1.28	.93 - 1.76	0.135	78.58%	<0.001	1.16	.64 - 2.09	0.630	76.12%	<0.001
	Yes	3	1.50	.78 - 2.91	0.228	91.09%	<0.001	2.01	.82 - 4.92	0.129	90.72%	<0.001	1.80	.26 - 12.65	0.557	92.60%	<0.001
Genotyping method	Array	5	0.96	.75 - 1.22	0.733	81.56%	<0.001	1.02	.77 - 1.36	0.881	77.78%	0.001	0.81	.49 - 1.34	0.407	75.91%	0.002
	RFLP	5	1.76	1.46 - 2.12	<0.001	0%	0.443	2.09	1.62 - 2.70	<0.001	10.23%	0.348	2.88	1.57 - 5.30	0.001	24.13%	0.266
Diagnosis of BC	Histologically	3	1.59	1.33 - 1.91	<0.001	0%	0.628	1.73	1.37 - 2.20	<0.001	0%	0.818	2.45	1.59 - 3.79	<0.001	0%	0.626
	Non-histologically	7	1.13	.87 - 1.47	0.355	83.45%	<0.001	1.31	.93 - 1.84	0.126	83.53%	<0.001	0.87	.51 - 1.48	0.605	72.74%	0.003
Sample size	Total sample size <300	5	1.50	.99 - 2.28	0.055	78.31%	0.001	1.87	1.20 - 2.91	0.005	63.90%	0.026	1.85	.51 - 6.68	0.347	84.43%	<0.001
	Total sample size >300	5	1.09	.84 - 1.40	0.525	84.67%	<0.001	1.15	.83 - 1.60	0.394	84.75%	<0.001	0.95	.61 - 1.48	0.806	66.82%	0.017
*ADIPOQ* rs2241766																	
Race	East Asian	1	1.26	.88 - 1.79	0.207	100%	NA	1.37	.90 - 2.08	0.139	100%	NA	1.11	.43 - 2.87	0.836	0%	NA
	Middle Eastern	3	1.36	.50 - 3.74	0.548	90.75%	<0.001	1.36	.43 - 4.32	0.602	90.66%	<0.001	1.56	.34 - 7.21	0.571	56.67%	0.099
	Others	2	0.53	.32 -.88	0.014	49.64%	0.159	0.55	.30 - 1.01	0.053	56.37%	0.130	0.20	.09 -.45	<0.001	0%	0.871
	Western	3	0.83	.67 - 1.03	0.093	41.25%	0.182	0.80	.62 - 1.05	0.103	49.41%	0.139	0.79	.49 - 1.27	0.331	0%	0.696
Study design	Prospective	2	0.95	.75 - 1.21	0.671	0%	0.696	0.94	.72 - 1.22	0.636	0%	0.759	0.97	.41 - 2.29	0.949	0%	0.529
	Retrospective	7	0.91	.62 - 1.35	0.653	86.82%	<0.001	0.92	.60 - 1.43	0.715	85.84%	<0.001	0.73	.34 - 1.59	0.430	65.55%	0.008
Control source	Hospital	7	0.91	.62 - 1.35	0.653	86.82%	<0.001	0.92	.60 - 1.43	0.715	85.84%	<0.001	0.73	.34 - 1.59	0.430	65.55%	0.008
	Population	2	0.95	.75 - 1.21	0.671	0%	0.696	0.94	.72 - 1.22	0.636	0%	0.759	0.97	.41 - 2.29	0.949	0%	0.529
Matched	NA	7	0.82	.63 - 1.08	0.168	68.69%	0.004	0.84	.63 - 1.12	0.228	64.68%	0.009	0.61	.31 - 1.20	0.151	42.14%	0.110
	Yes	2	1.60	.32 - 8.02	0.571	96.43%	<0.001	1.60	.271 - 9.411	0.606	96.20%	<0.001	1.87	.23 - 15.49	0.564	84.78%	0.010
Genotyping method	Array	5	0.75	.63 -.90	0.001	39.40%	0.159	0.74	.61 -.89	0.001	32.69%	0.203	0.60	.31 - 1.15	0.122	51.87%	0.081
	RFLP	4	1.21	.58 - 2.56	0.612	87.81%	<0.001	1.29	.58 - 2.86	0.529	86.27%	<0.001	1.22	.34 - 4.46	0.761	53.34%	0.092
Diagnosis of BC	Histologically	4	1.00	.43 - 2.34	0.999	91.82%	<0.001	1.07	.45 - 2.53	0.883	89.92%	<0.001	0.62	.09 - 4.29	0.632	79.72%	0.002
	Non-histologically	5	0.88	.69 - 1.11	0.278	61.51%	0.034	0.85	.63 - 1.15	0.286	69.24%	0.011	0.84	.56 - 1.26	0.402	0%	0.891
Sample size	Total sample size <300	4	1.00	.40 - 2.51	0.992	90.38%	<0.001	0.99	.36 - 2.75	0.985	89.95%	<0.001	1.06	.24 - 4.63	0.938	55.92%	0.078
	Total sample size >300	5	0.85	.68 - 1.08	0.185	68.91%	0.012	0.86	.67 - 1.11	0.239	65.31%	0.021	0.66	.34 - 1.26	0.209	58.73%	0.046

R, risk allele; W, wild allele; OR, odds ratio; 95% CI, 95% confidence interval; NA, not available.

### Sensitivity analyses

Sensitivity analyses were performed for 5 genetic alterations associated with breast cancer under allele mode of inheritance, respectively (**Supplementary Figure 3**). There was no observably significant impact of any individual studies on overall effect-size estimates for 5 genetic alterations evaluated.

### Publication bias

**Figure 3:**
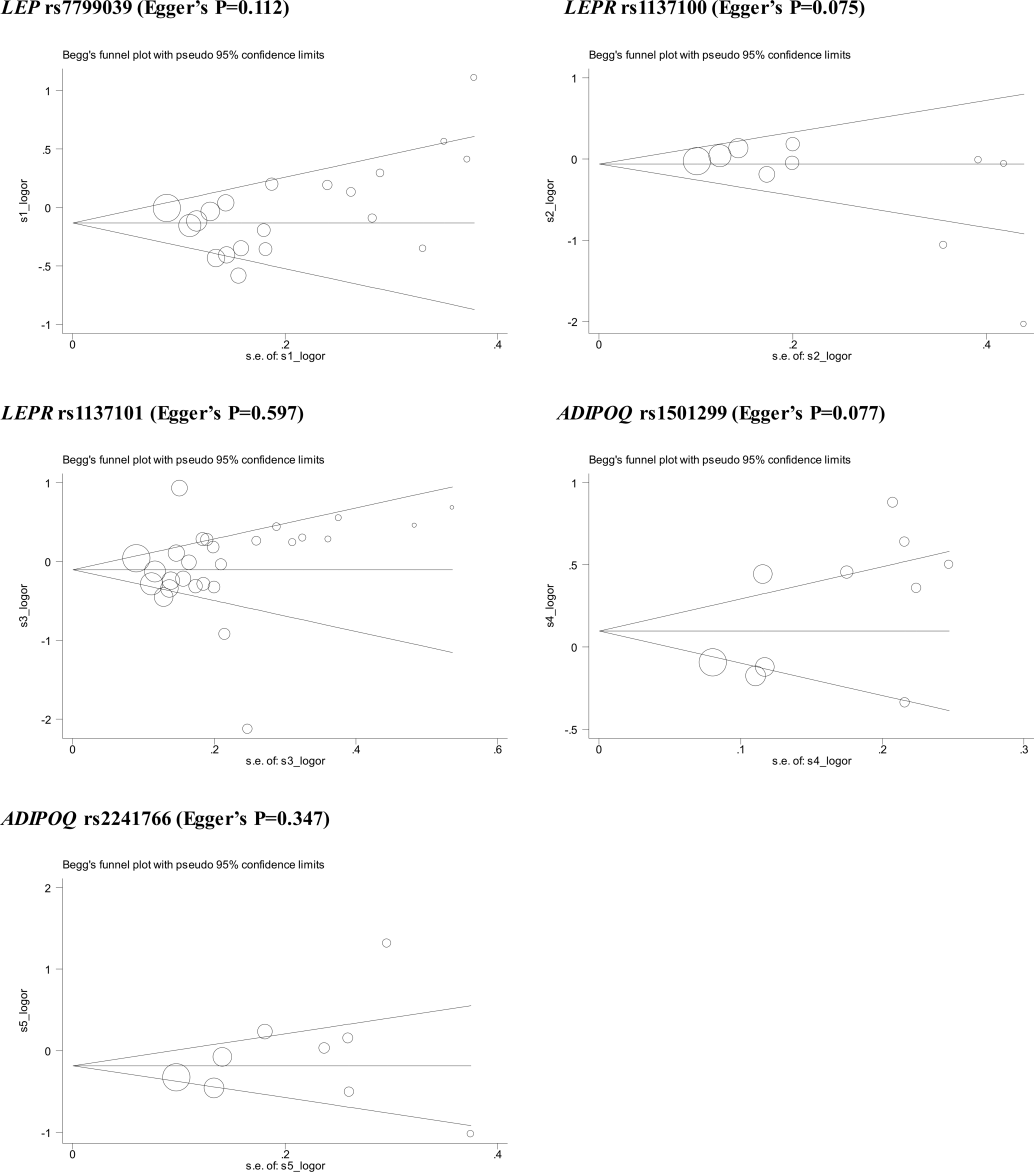
Begg’s funnel plots of 5 genetic alterations associated with breast cancer under allele mode of inheritance.

Publication bias was assessed in the form of funnel plots and regression tests. As shown in **Figure 3**, Begg’s funnel plots seemed symmetrical, with the exception of *LEPR* gene rs1137100 and *ADIPOQ* gene rs1501299, which was confirmed by the Egger’s regression tests (P: 0.075 and 0.077, respectively). Filled funnel plots revealed that two studies and one study were theoretically missing for *LEPR* gene rs1137100 and *ADIPOQ* gene rs1501299, respectively. After taking these missing studies into consideration, effect-size estimates were changed slightly.

### Circulating leptin levels

**Figure 4:**
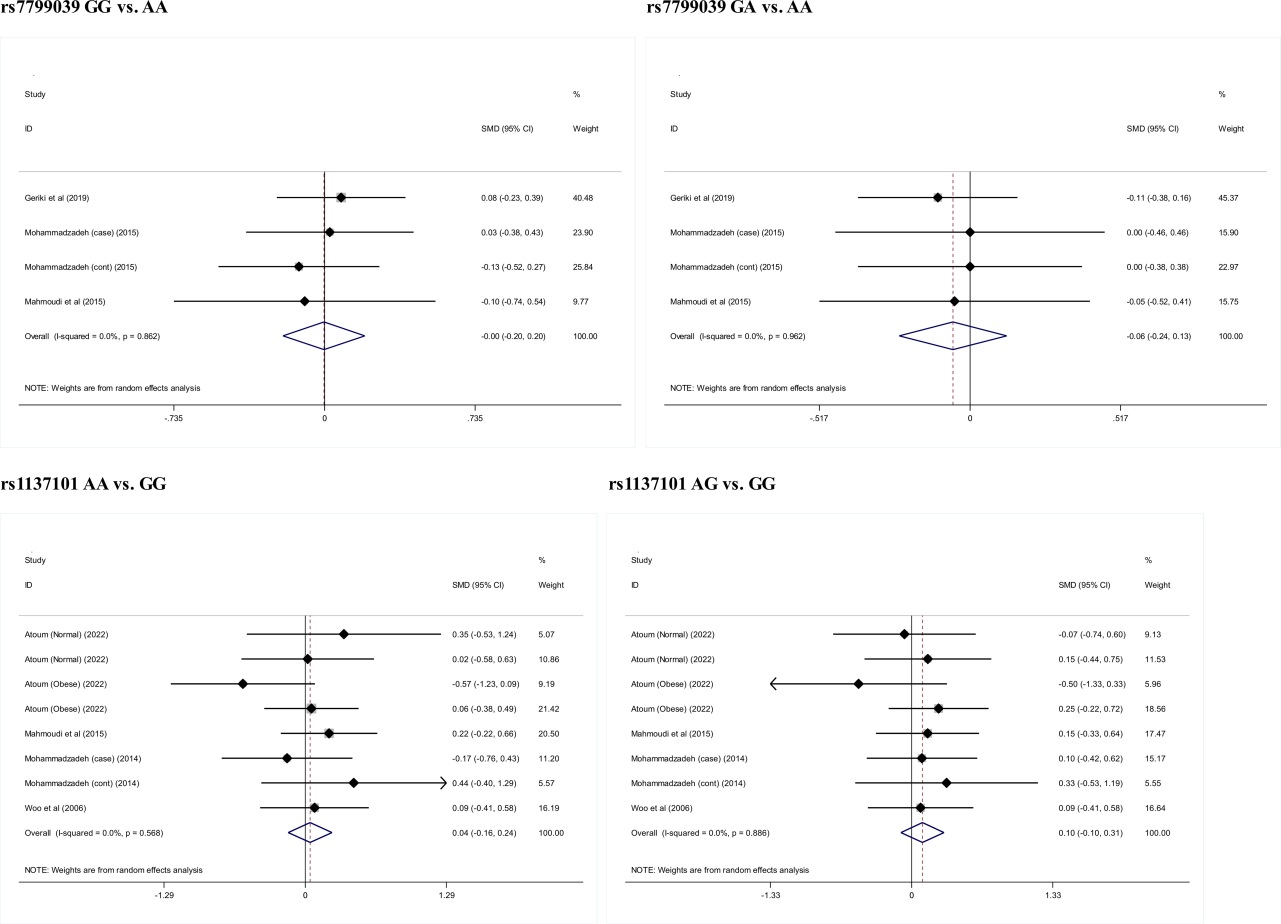
Changes of circulating leptin levels across genotypes of rs7799039 and rs1137101.

**Figure 4** presents the comparison of circulating leptin levels between genotypes of *LEP* gene rs7799039 and *LEPR* gene rs1137101. There was no noticeable difference for all comparisons (P>0.05).

## Discussion

The aim of this meta-analysis was to examine the association of 5 genetic alterations in *LEP* and *ADIPOQ* genes, as well as their receptor-encoded genes, with breast cancer risk and circulating leptin levels. Importantly, we found that *LEP* gene rs7799039 and *ADIPOQ* gene rs1501299 were two promising candidate loci in predisposition to breast cancer risk. Additionally, we found that differences in menopausal status, obesity, race, study design, diagnosis of breast cancer, genotyping method and sample size might account for the divergent results of previous studies in the literature. To the best of our knowledge, this meta-analysis is thus far the most comprehensive on the susceptibility of *LEP* and *ADIPOQ* genes to breast cancer.

Breast cancer has a strong genetic predisposition, and the heritability among first degree relatives is estimated to be around 35% (51, 52). To unravel the genetic linings of breast cancer, a large panel of studies have been conducted, and many genes were identified to be susceptible to breast cancer, such as breast cancer susceptibility gene (*BRCA*) (53). However, for the majority of identified genes, uncertainty still exists over which gene is actually involved in the pathogenesis of breast cancer. One of the biggest hurdles is lack of reproducibility across single studies. The reasons for this irreproducibility are mainly attributed to insufficient power to detect significance, discrepant sampling criteria of participants and varying characteristics of participants. Taking these possible reasons into consideration, we in this meta-analysis tested the hypothesis that genes encoding leptin and adiponectin and their receptors are potential candidates to breast cancer. Our findings supported this hypothesis by showing that *LEP* gene rs7799039 and *ADIPOQ* gene rs1501299 were two promising breast cancer-susceptibility loci. By contrast, a recent meta-analysis by Sayad and coworkers did not support the association of *LEP* gene rs7799039 and *LEPR* gene rs1137100 with breast cancer (54). It is possibly because of the differing number of eligible studies involved between the meta-analysis by Sayad and coworkers (54) and the present meta-analysis. As far as we know, we, for the first time, meta-analyzed the association between *ADIPOQ* gene and breast cancer.

To seek possible reasons behind the irreproducible findings of previous studies, we further conducted subgroup analyses for the association of 5 genetic alterations with breast cancer under three genetic modes of inheritance. It is of importance to see that menopausal status, obesity and race were potential attributes responsible for this irreproducibility. The impact of menopausal status and obesity on breast carcinogenesis has been well established, with evidence from both clinical and experimental aspects (55–57). The attribute race merited special discussion, as it is not uncommon to notice that a genetic alteration is associated with a disease in one racial group but not in another (58, 59). Given that linkage disequilibrium and genetic sequences may not be identical across races, it is a wise choice to establish candidate genes and genetic alterations within each race or ethnicity group.

Although the significant association between *LEP*, *LEPR* and *ADIPOQ* genes and breast cancer risk, we did not notice remarkable differences in circulating leptin levels across genotypes of their genetic alterations. The possibility for this phenomenon might be the limited number of studies measuring and comparing circulating leptin levels across genotypes. Another possibility is that studies for the association with breast cancer and circulating leptin levels are not identical, and differences in study designs, sample sizes and participant characteristics may matter. Practically, it is expected to validate the association with circulating leptin levels by large, well-designed cohorts in the future.

### Limitations

Some possible limitations needed to be addressed for this meta-analysis. The first is the probability of selection bias. This meta-analysis merely retrieved published studies in English, and studies written the other languages known as “grey” literature were not covered. The second limitation is the cross-sectional nature of all retrieved studies, and the association derived in this meta-analysis cannot imply the cause-and-effect relationship, calling for further investigations to fill this gap in knowledge. The third limitation is the insufficient power in most subgroup association analyses. The fourth limitation is that this meta-analysis is based on summary estimates, instead of individual participant data, which made the statistical correction for some confounding factors such as menopausal status and body mass index impractical. The fifth limitation is the possibility of publication bias for two of five genetic alterations assessed in this meta-analysis; however, incorporation of adding theoretically missing studies did not materially change our effect-size estimates.

In conclusion, through a comprehensive analysis of 33 publications, we found that *LEP* gene rs7799039 and *ADIPOQ* gene rs1501299 were two promising candidate loci in predisposition to breast cancer risk. Additionally, we found that differences in menopausal status, obesity, race, study design, diagnosis of breast cancer, genotyping method and sample size might account for the divergent results of previous studies in the literature. We agree that further investigations from genetic and experimental points of view are necessary to ascertain the implication of these genes in the pathogenesis of breast cancer, which might shed more light on knowledge and preferences toward breast cancer screening for high-risk women.

## Data availability statement

The original contributions presented in the study are included in the article/**Supplementary Material.** Further inquiries can be directed to the corresponding author.

## Author contributions

W-zP conceived the study; XL and C-fL conducted the literature search. XL and C-fL extracted the required data. XL and C-fL performed data analysis and interpretation. W-zP and JZ did statistical analyses. W-zP and XL drafted the manuscript. All authors contributed to the writings and approved the final version of the manuscript.

## References

[1] Siegel RL Miller KD Fuchs HE Jemal A . Cancer statistics, 2022. CA Cancer J Clin (2022) 72(1):7–33. doi: 10.3322/caac.21708 35020204

[2] Giaquinto AN Sung H Miller KD Kramer JL Newman LA Minihan A . Breast cancer statistics, 2022. CA Cancer J Clin (2022) 72(6):524–41. doi: 10.3322/caac.21754 36190501

[3] Criscitiello C Corti C . Breast cancer genetics: diagnostics and treatment. Genes (Basel) (2022) 13(9). doi: 10.3390/genes13091593 PMC9498728 36140761

[4] Lynch HT Harris RE Organ CH Jr. Lynch JF . Management of familial breast cancer. i. biostatistical-genetic aspects and their limitations as derived from a familial breast cancer resource. Arch Surg (1978) 113(9):1053–8. doi: 10.1001/archsurg.1978.01370210035004 687103

[5] Morton LM Sampson JN Armstrong GT Chen TH Hudson MM Karlins E . Genome-wide association study to identify susceptibility loci that modify radiation-related risk for breast cancer after childhood cancer. J Natl Cancer Inst (2017) 109(11). doi: 10.1093/jnci/djx058 PMC6059172 29059430

[6] Michailidou K Beesley J Lindstrom S Canisius S Dennis J Lush MJ . Genome-wide association analysis of more than 120,000 individuals identifies 15 new susceptibility loci for breast cancer. Nat Genet (2015) 47(4):373–80. doi: 10.1038/ng.3242 PMC4549775 25751625

[7] Cai Q Zhang B Sung H Low SK Kweon SS Lu W . Genome-wide association analysis in East asians identifies breast cancer susceptibility loci at 1q32. 1 5q14.3 15q26.1. Nat Genet (2014) 46(8):886–90. doi: 10.1038/ng.3041 PMC4127632 25038754

[8] Orr N Lemnrau A Cooke R Fletcher O Tomczyk K Jones M . Genome-wide association study identifies a common variant in RAD51B associated with male breast cancer risk. Nat Genet (2012) 44(11):1182–4. doi: 10.1038/ng.2417 PMC3722904 23001122

[9] Turnbull C Ahmed S Morrison J Pernet D Renwick A Maranian M . Genome-wide association study identifies five new breast cancer susceptibility loci. Nat Genet (2010) 42(6):504–7. doi: 10.1038/ng.586 PMC3632836 20453838

[10] Geriki S Bitla AR SrinivasaRao P Hulikal N Yootla M Sachan A . Association of single nucleotide polymorphisms of adiponectin and leptin genes with breast cancer. Mol Biol Rep (2019) 46(6):6287–97. doi: 10.1007/s11033-019-05070-5 31538300

[11] Sabol RA Bowles AC Cote A Wise R O'Donnell B Matossian MD . Leptin produced by obesity-altered adipose stem cells promotes metastasis but not tumorigenesis of triple-negative breast cancer in orthotopic xenograft and patient-derived xenograft models. Breast Cancer Res (2019) 21(1):67. doi: 10.1186/s13058-019-1153-9 31118047 PMC6530039

[12] Perera CN Spalding HS Mohammed SI Camarillo IG . Identification of proteins secreted from leptin stimulated MCF-7 breast cancer cells: a dual proteomic approach. Exp Biol Med (Maywood). (2008) 233(6):708–20. doi: 10.3181/0710-RM-281 18408141

[13] Romero-Figueroa Mdel S Garduno-Garcia Jde J Duarte-Mote J Matute-Gonzalez G Gomez-Villanueva A de la Cruz-Vargas J . Insulin and leptin levels in obese patients with and without breast cancer. Clin Breast Cancer (2013) 13(6):482–5. doi: 10.1016/j.clbc.2013.08.001 24084031

[14] Chung SJ Nagaraju GP Nagalingam A Muniraj N Kuppusamy P Walker A . ADIPOQ/adiponectin induces cytotoxic autophagy in breast cancer cells through STK11/LKB1-mediated activation of the AMPK-ULK1 axis. Autophagy (2017) 13(8):1386–403. doi: 10.1080/15548627.2017.1332565 PMC5584870 28696138

[15] Bielawski K Rhone P Bulsa M Ruszkowska-Ciastek B . Pre-operative combination of normal BMI with elevated YKL-40 and leptin but lower adiponectin level is linked to a higher risk of breast cancer relapse: a report of four-year follow-up study. J Clin Med (2020) 9(6). doi: 10.3390/jcm9061742 PMC7355849 32512860

[16] Grossmann ME Cleary MP . The balance between leptin and adiponectin in the control of carcinogenesis - focus on mammary tumorigenesis. Biochimie (2012) 94(10):2164–71. doi: 10.1016/j.biochi.2012.06.013 PMC4296518 22728769

[17] Sultana R Kataki AC Borthakur BB Basumatary TK Bose S . Imbalance in leptin-adiponectin levels and leptin receptor expression as chief contributors to triple negative breast cancer progression in northeast India. Gene (2017) 621:51–8. doi: 10.1016/j.gene.2017.04.021 28414093

[18] Moher D Liberati A Tetzlaff J Altman DG Group P . Preferred reporting items for systematic reviews and meta-analyses: the PRISMA statement. PloS Med (2009) 6(7):e1000097. doi: 10.1371/journal.pmed.1000097 19621072 PMC2707599

[19] Li L Meng X Liu L Xiang Y Wang F Yu L . Single-nucleotide polymorphisms in LEP and LEPR associated with breast cancer risk: results from a multicenter case-control study in Chinese females. Front Oncol (2022) 12:809570. doi: 10.3389/fonc.2022.809570 35223490 PMC8866686

[20] Atoum MF Hamaid Alparrey AA . Association of leptin receptor Q223R gene polymorphism and breast cancer patients: a case control study. Asian Pac J Cancer Prev (2022) 23(1):177–82. doi: 10.31557/APJCP.2022.23.1.177 PMC9258657 35092386

[21] Özgöz A Mutlu Içduygu F Yükseltürk A Samli H Hekimler Öztürk K Baskan Z . Postmenopausal estrogen receptor positive breast cancer and obesity associated gene variants. Excli J (2021) 20:1133–44. doi: 10.17179/excli2020-2860 PMC8326496 34345232

[22] Hołysz H Paszel-Jaworska A Romaniuk-Drapała A Grodecka-Gazdecka S Rubiś B . LEP (-2548G>A LEP) and LEPR (223Gln>Arg, 109Lys>Arg) polymorphisms as breast cancer risk factors in the polish female population. Mol Biol Rep (2021) 48(4):3237–44. doi: 10.1007/s11033-021-06328-7 PMC8172510 33864589

[23] Mahmoud EH Fawzy A El-Din WM Shafik NF . Diagnostic value of adiponectin gene polymorphism and serum level in postmenopausal obese patients with breast cancer. J Cancer Res Ther (2020) 16(6):1269–73. doi: 10.4103/jcrt.JCRT_1091_19 33342783

[24] Cerda-Flores RM Camarillo-Cárdenas KP Gutiérrez-Orozco G Villarreal-Vela MP Garza-Guajardo R Ponce-Camacho MA . ADIPOQ single nucleotide polymorphisms and breast cancer in northeastern Mexican women. BMC Med Genet (2020) 21(1):187. doi: 10.1186/s12881-020-01125-8 32977760 PMC7519484

[25] Pasha HF Mohamed RH Toam MM Yehia AM . Genetic and epigenetic modifications of adiponectin gene: potential association with breast cancer risk. J Gene Med (2019) 21(10):e3120. doi: 10.1002/jgm.3120 31415715

[26] Macias-Gomez NM Hernandez-Terrones MC Ramirez-Guerrero AA Leal-Ugarte E Gutierrez-Angulo M Peregrina-Sandoval J . ADIPOQ rs2241766 SNP as protective marker against DIBC development in Mexican population. PloS One (2019) 14(3):e0214080. doi: 10.1371/journal.pone.0214080 30883598 PMC6422300

[27] Liu CR Li Q Hou C Li H Shuai P Zhao M . Changes in body mass index, leptin, and leptin receptor polymorphisms and breast cancer risk. DNA Cell Biol (2018) 37(3):182–8. doi: 10.1089/dna.2017.4047 29336592

[28] Rodrigo C Tennekoon KH Karunanayake EH De Silva K Amarasinghe I Wijayasiri A . Circulating leptin, soluble leptin receptor, free leptin index, visfatin and selected leptin and leptin receptor gene polymorphisms in sporadic breast cancer. Endocr J (2017) 64(4):393–401. doi: 10.1507/endocrj.EJ16-0448 28190851

[29] El-Hussiny MA Atwa MA Rashad WE Shaheen DA Elkady NM . Leptin receptor Q223R polymorphism in Egyptian female patients with breast cancer. Contemp Oncol (Pozn) (2017) 21(1):42–7. doi: 10.5114/wo.2017.66655 PMC5385477 28435397

[30] Khandouzi M Deka M Narayan Baruah M Rozati R Kordafshari M Kashyap P . Genetic variation in adiponectin, leptin and leptin receptors with reference to risk of breast cancer in northeast obese women in India. BIOSCIENCE Biotechnol Res Commun (2016) 9(2):163–70. doi: 10.21786/bbrc/9.2/1

[31] Erbay B Yılmaz TU Eraldemir C Üren N Tiryaki Ç Ergül E . The relationship between adiponectin and breast cancer. J Breast Health (2016) 12(2):67–71. doi: 10.5152/tjbh.2016.2881 28331736 PMC5351503

[32] Rostami S Kohan L Mohammadianpanah M . The LEP G-2548A gene polymorphism is associated with age at menarche and breast cancer susceptibility. Gene (2015) 557(2):154–7. doi: 10.1016/j.gene.2014.12.021 25510398

[33] Mohammadzadeh G Ghaffari MA Bafandeh A Hosseini SM Ahmadi B . The relationship between -2548 G/A leptin gene polymorphism and risk of breast cancer and serum leptin levels in ahvazian women. Iran J Cancer Prev (2015) 8(2):100–8. PMC4411471 25960849

[34] Mahmoudi R Noori Alavicheh B Nazer Mozaffari MA Fararouei M Nikseresht M . Polymorphisms of leptin (-2548 G/A) and leptin receptor (Q223R) genes in Iranian women with breast cancer. Int J Genomics (2015) 2015:132720. doi: 10.1155/2015/132720 26199932 PMC4496654

[35] Karakus N Kara N Ulusoy AN Ozaslan C Tural S Okan I . Evaluation of CYP17A1 and LEP gene polymorphisms in breast cancer. Oncol Res Treat (2015) 38(9):418–22. doi: 10.1159/000438940 26407154

[36] Mohammadzadeh G Ghaffari MA Bafandeh A Hosseini SM . Effect of leptin receptor Q223R polymorphism on breast cancer risk. Iran J Basic Med Sci (2014) 17(8):588–94. PMC4240793 25422752

[37] Robles MJG . The LEP G-2548A polymorphism is not associated with breast cancer susceptibility in obese Western Mexican women. J Clin Cell Immunol (2013) 04(01). doi: 10.4172/2155-9899.1000133

[38] Kaklamani VG Hoffmann TJ Thornton TA Hayes G Chlebowski R Van Horn L . Adiponectin pathway polymorphisms and risk of breast cancer in African americans and hispanics in the women's health initiative. Breast Cancer Res Treat (2013) 139(2):461–8. doi: 10.1007/s10549-013-2546-6 PMC3773607 23624817

[39] Kim KZ Shin A Lee YS Kim SY Kim Y Lee ES . Polymorphisms in adiposity-related genes are associated with age at menarche and menopause in breast cancer patients and healthy women. Hum Reprod (2012) 27(7):2193–200. doi: 10.1093/humrep/des147 22537818

[40] Gu F Kraft P Rice M Michels KB . Leptin and leptin receptor genes in relation to premenopausal breast cancer incidence and grade in Caucasian women. Breast Cancer Res Treat (2012) 131(1):17–25. doi: 10.1007/s10549-011-1778-6 21947707 PMC3627481

[41] Nyante SJ Gammon MD Kaufman JS Bensen JT Lin DY Barnholtz-Sloan JS . Common genetic variation in adiponectin, leptin, and leptin receptor and association with breast cancer subtypes. Breast Cancer Res Treat (2011) 129(2):593–606. doi: 10.1007/s10549-011-1517-z 21516303 PMC3355661

[42] Cleveland RJ Gammon MD Long CM Gaudet MM Eng SM Teitelbaum SL . Common genetic variations in the LEP and LEPR genes, obesity and breast cancer incidence and survival. Breast Cancer Res Treat (2010) 120(3):745–52. doi: 10.1007/s10549-009-0503-1 PMC3571680 19697123

[43] Teras LR Goodman M Patel AV Bouzyk M Tang W Diver WR . No association between polymorphisms in LEP, LEPR, ADIPOQ, ADIPOR1, or ADIPOR2 and postmenopausal breast cancer risk. Cancer Epidemiol Biomarkers Prev (2009) 18(9):2553–7. doi: 10.1158/1055-9965.EPI-09-0542 19723917

[44] Okobia MN Bunker CH Garte SJ Zmuda JM Ezeome ER Anyanwu SN . Leptin receptor Gln223Arg polymorphism and breast cancer risk in Nigerian women: a case control study. BMC Cancer (2008) 8:338. doi: 10.1186/1471-2407-8-338 19017403 PMC2613914

[45] Kaklamani VG Sadim M Hsi A Offit K Oddoux C Ostrer H . Variants of the adiponectin and adiponectin receptor 1 genes and breast cancer risk. Cancer Res (2008) 68(9):3178–84. doi: 10.1158/0008-5472.CAN-08-0533 PMC2685173 18451143

[46] Han CZ Du LL Jing JX Zhao XW Tian FG Shi J . Associations among lipids, leptin, and leptin receptor gene Gin223Arg polymorphisms and breast cancer in China. Biol Trace Elem Res (2008) 126(1-3):38–48. doi: 10.1007/s12011-008-8182-z 18668212

[47] Liu CL Chang YC Cheng SP Chern SR Yang TL Lee JJ . The roles of serum leptin concentration and polymorphism in leptin receptor gene at codon 109 in breast cancer. Oncology (2007) 72(1-2):75–81. doi: 10.1159/000111097 18004080

[48] Gallicchio L McSorley MA Newschaffer CJ Huang HY Thuita LW Hoffman SC . Body mass, polymorphisms in obesity-related genes, and the risk of developing breast cancer among women with benign breast disease. Cancer Detect Prev (2007) 31(2):95–101. doi: 10.1016/j.cdp.2007.02.004 17428620

[49] Woo HY Park H Ki CS Park YL Bae WG . Relationships among serum leptin, leptin receptor gene polymorphisms, and breast cancer in Korea. Cancer Lett (2006) 237(1):137–42. doi: 10.1016/j.canlet.2005.05.041 16011872

[50] Snoussi K Strosberg AD Bouaouina N Ben Ahmed S Helal AN Chouchane L . Leptin and leptin receptor polymorphisms are associated with increased risk and poor prognosis of breast carcinoma. BMC Cancer. (2006) 6:38. doi: 10.1186/1471-2407-6-38 16504019 PMC1397853

[51] Ju W Wang J Li B Li Z . An epidemiology and molecular genetic study on breast cancer susceptibility. Chin Med Sci J (2000) 15(4):231–7. 12906145

[52] Calvo Chozas A Mahjani B Ronnegard L . Family history of breast cancer is associated with elevated risk of prostate cancer: evidence for shared genetic risks. Hum Hered (2021). doi: 10.1159/000521215 34847553

[53] Osorio A de la Hoya M Rodriguez-Lopez R Martinez-Ramirez A Cazorla A Granizo JJ . Loss of heterozygosity analysis at the BRCA loci in tumor samples from patients with familial breast cancer. Int J Cancer (2002) 99(2):305–9. doi: 10.1002/ijc.10337 11979449

[54] Sayad S Dastgheib SA Farbod M Asadian F Karimi-Zarchi M Salari S . Association of PON1, LEP and LEPR polymorphisms with susceptibility to breast cancer: a meta-analysis. Asian Pac J Cancer Prev (2021) 22(8):2323–34. doi: 10.31557/APJCP.2021.22.8.2323 PMC8629481 34452542

[55] Crujeiras AB Diaz-Lagares A Stefansson OA Macias-Gonzalez M Sandoval J Cueva J . Obesity and menopause modify the epigenomic profile of breast cancer. Endocr Relat Cancer (2017) 24(7):351–63. doi: 10.1530/ERC-16-0565 28442560

[56] Katoh A Watzlaf VJ D'Amico F . An examination of obesity and breast cancer survival in post-menopausal women. Br J Cancer (1994) 70(5):928–33. doi: 10.1038/bjc.1994.422 PMC2033566 7947099

[57] Garcia-Estevez L Cortes J Perez S Calvo I Gallegos I Moreno-Bueno G . Obesity and breast cancer: a paradoxical and controversial relationship influenced by menopausal status. Front Oncol (2021) 11:705911. doi: 10.3389/fonc.2021.705911 34485137 PMC8414651

[58] Sweeney C Bernard PS Factor RE Kwan ML Habel LA Quesenberry CP Jr. . Intrinsic subtypes from PAM50 gene expression assay in a population-based breast cancer cohort: differences by age, race, and tumor characteristics. Cancer Epidemiol Biomarkers Prev (2014) 23(5):714–24. doi: 10.1158/1055-9965.EPI-13-1023 PMC4011983 24521995

[59] Chavez-Macgregor M Liu S De Melo-Gagliato D Chen H Do KA Pusztai L . Differences in gene and protein expression and the effects of race/ethnicity on breast cancer subtypes. Cancer Epidemiol Biomarkers Prev (2014) 23(2):316–23. doi: 10.1158/1055-9965.EPI-13-0929 PMC3946290 24296856

